# Supplementary material for: Neuroblastoma Tumor-Associated Mesenchymal Stromal Cells Regulate the Cytolytic Functions of NK Cells
Source: Cancers (Basel). 2022 Dec 20;15(1):19. doi: 10.3390/cancers15010019 (PMC9818020; doi:10.3390/cancers15010019)
Supplement: Supplementary file 1 [file cancers-15-00019-s001.zip › cancers-2070183-supplementary.pdf]

Supplementary Figure S1.

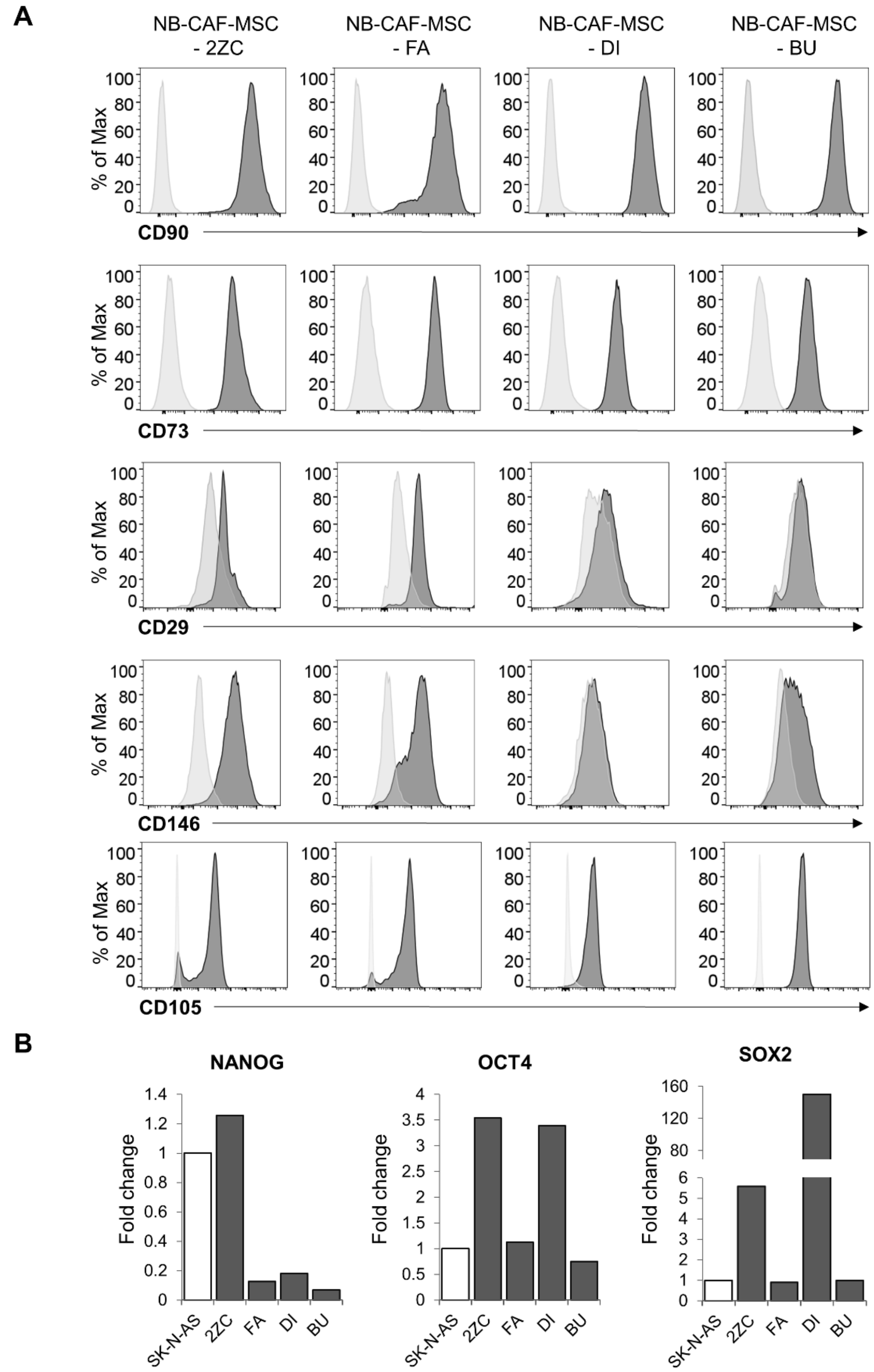

Supplementary Figure S1. Evaluation of the Mesenchymal phenotype in primary NB-TA-MSC cultures. (**A**) Flow-cytometry analysis of the indicated surface markers (CD105, CD90, CD73, CD29 and CD146). Light grey histograms represent unstained control; dark grey histograms represent stained samples. A representative experiment is shown of *n* = 5 experiments performed. (**B**) RT-qPCR analysis

of SOX2, NANOG and OCT4 stemness genes transcript in different primary NB-TA-MSC cultures. The NB commercial cell line SK-N-AS was used as reference control. Histograms represent the fold change of gene transcript expression normalized for GAPDH expression compared to SK-N-AS expression which level is arbitrarily set as 1. Data are expressed as mean ± SD ($n = 3$).

Supplementary Figure S2.

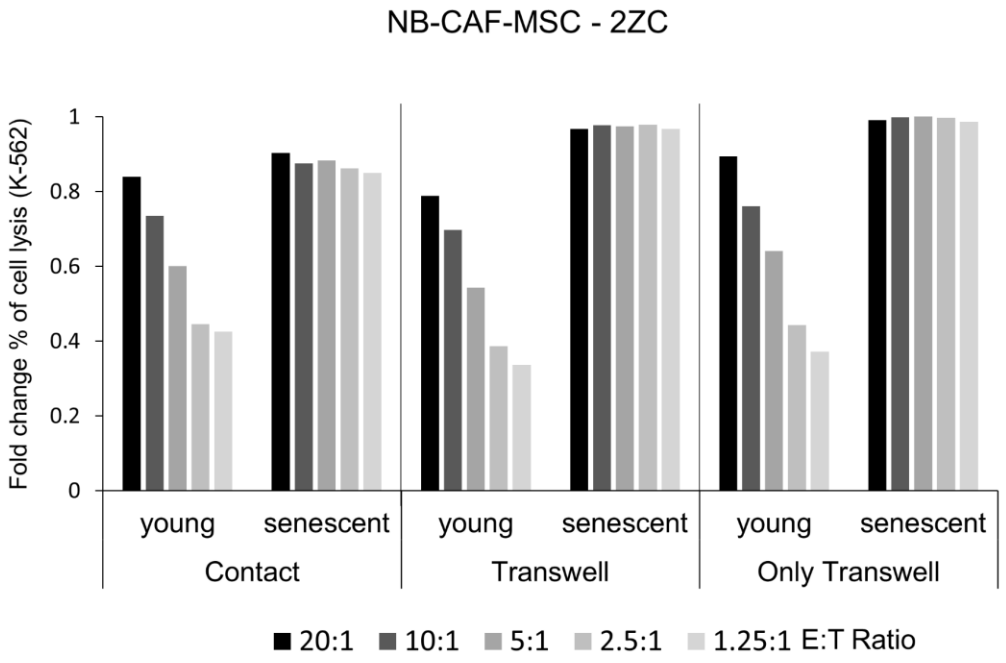

Supplementary Figure S2. Senescent NB-CAF-MSC – 2ZC lose their immunomodulatory properties in comparison to the young cells. Percentage of K-562 cells lysis in cytotoxicity assays using freshly isolated NK cells after co-culture with young and senescent NB-CAF-MSC – 2ZC primary cultures under direct cell-cell contact or under Transwell conditions. Data presented here are expressed as fold change of percentage of cell lysis vs CTRL.
